# A Model of Post-Infection Fatigue Is Associated with Increased TNF and 5-HT_2A_ Receptor Expression in Mice

**DOI:** 10.1371/journal.pone.0130643

**Published:** 2015-07-06

**Authors:** Yvonne Couch, Qin Xie, Louise Lundberg, Trevor Sharp, Daniel C. Anthony

**Affiliations:** 1 Department of Pharmacology, Mansfield Road, Oxford, OX1 3QT, United Kingdom; 2 Public Health England, Centre for Radiation, Chemical and Environmental Hazards, Chilton, Didcot, Oxford OX11 0RQ, United Kingdom; Universidade do Estado do Rio de Janeiro, BRAZIL

## Abstract

It is well documented that serotonin (5-HT) plays an important role in psychiatric illness. For example, myalgic encephalomyelitis (ME/CFS), which is often provoked by infection, is a disabling illness with an unknown aetiology and diagnosis is based on symptom-specific criteria. However, 5-HT_2A_ receptor expression and peripheral cytokines are known to be upregulated in ME. We sought to examine the relationship between the 5-HT system and cytokine expression following systemic bacterial endotoxin challenge (LPS, 0.5mg/kg i.p.), at a time when the acute sickness behaviours have largely resolved. At 24 hours post-injection mice exhibit no overt changes in locomotor behaviour, but do show increased immobility in a forced swim test, as well as decreased sucrose preference and reduced marble burying activity, indicating a depressive-like state. While peripheral IDO activity was increased after LPS challenge, central activity levels remained stable and there was no change in total brain 5-HT levels or 5-HIAA/5-HT. However, within the brain, levels of TNF and 5-HT_2A_ receptor mRNA within various regions increased significantly. This increase in receptor expression is reflected by an increase in the functional response of the 5-HT_2A_ receptor to agonist, DOI. These data suggest that regulation of fatigue and depressive-like moods after episodes of systemic inflammation may be regulated by changes in 5-HT receptor expression, rather than by levels of enzyme activity or cytokine expression in the CNS.

## Introduction

Diseases such as myalgic encephalomyelitis/chronic fatigue syndrome (ME/CFS) are often provoked by infection and have been shown to be associated with altered expression of peripheral pro-inflammatory cytokines. Indeed, cytokine expression is higher in patients with ME/CFS than in individuals suffering from major depression, which is often argued to have a strong cytokine component [1]. The change in expression of 5-HT receptors, such as 5HT_2A_, is also a feature of fatigue, which suggests that dysregulation of the serotonergic system may also play a role in post-infection-associated behaviours [2]. Post-infection ME/CFS-like behaviours can be modeled by the injection of bacterial endotoxin (LPS). The metabolic response and decreased motility that usually accompany LPS administration quickly resolve, but many depression-like behaviours persist beyond the acute phase. The molecular mechanisms that underlie these behavioural changes in both humans and animals are complex and poorly understood. A popular theory argues that circulating cytokines such as tumor necrosis factor (TNF) and interleukin(IL)-1β increase the activity of the enzyme indoleamine 2,3-dioxygenase (IDO) within the brain which reduces the availability of tryptophan, a precursor for 5-HT synthesis [3] and thus the overall levels of 5-HT in the CNS. However, the peripheral injection of *M*.*Bovis* BCG has been shown to not alter the CNS kynurenine/tryptophan ratio [4], which is reported to be an indirect measure of IDO activity and therefore it is unclear whether this is solely a peripheral phenomenon, or whether IDO activity in the brain is important in regulating these behaviours.

An alternative hypothesis to the IDO theory is that the behaviours may be dependent on post-synaptic changes, rather than changes in 5-HT release or overall levels of 5-HT in the brain. We have previously shown that there is no significant decrease in brain 5-HT in a model of systemic inflammation [5], but that there is an increase in the expression certain 5-HT receptors in the CNS and that this change is related to a functional outcome. Animal models of depression also frequently show altered receptor expression levels, even when the genetic manipulation is in an unrelated gene. Pang and colleagues found altered levels of 5-HT_1B_ and 5-HT_2A_ receptors in the hippocampus and cortex in a model of Huntington’s after observing depression-like behaviour [6]. Both Maes [7] and Dantzer [8] suggest that sickness behaviour is an acute response, characterized by similar behavioural phenomenology as depression but with a pyretic component, and that behavioural responses persisting after 24 hours should be considered depression, rather than sickness.

Currently, the literature remains unclear as to whether the persistent behavioural changes that result from systemic inflammatory challenges are the result of systemic cytokines or CNS cytokines. Work studying the behavioural changes in response to inflammatory challenges often focuses on the role of cytokines in the CNS, usually TNF and IL-1β. There is significant evidence that both TNF and IL-1β play important roles in modulating behaviour [9, 10] but controversy still exists over the relative contributions of one cytokine vs the other [9]. We have demonstrated previously that there is significant up-regulation of TNF in the prefrontal cortex of animals defined as behaviourally ‘depressed’ in a model of chronic stress, and that this may be associated with changes in the 5-HT system [11].

With these facts in mind, we aimed to study LPS-induced fatigue symptoms in the mouse 24 hours after a systemic challenge, directly measuring tryptophan breakdown and 5-HT levels in the CNS, in addition to cytokine and 5-HT receptor expression, in order to establish whether there is a correlation between the 5-HT system and LPS-induced sickness behaviours. Our results suggest that some behavioural changes associated with CFS/ME-like depressive behaviour may occur independently of CNS tryptophan levels but rather may be the result of changes in 5-HT receptor expression within the CNS.

## Materials and Methods

### Animals

Adult, outbred, male CD-1 mice (8 weeks) were obtained from Harlan (UK) and housed under a standard 12 hour light/dark cycle and provided with food/water *ad libitum*. Animals were housed in groups of 4 in a standard open cage with sawdust substrate and enrichment (card house, plastic tube, nesting material). Animals were from mixed litters and were housed with conspecifics, rather than littermates. Allocation to LPS/vehicle (saline) treatment was random. All procedures were carried out in accordance with the UK Animals (Scientific Procedures) Act, 1986. The protocols were carried out with approval of local ethical committees (University of Oxford, Clinical Medicine AWERB) and all efforts were made to minimise pain and suffering. Animals were allowed to acclimatize to housing conditions for 1 week before any testing was carried out. Peripheral LPS challenge constituted one i.p. injection of LPS (026:B6; Sigma, UK) at 0.5mg/kg. All animals were healthy at the time of injection.

### Behaviour

All testing was carried out between the hours of 10:00 and 16:00 of the light phase of the light/dark cycle. Light levels within home cages were 5–8 Lux. In order to avoid differences in odour cues from one mouse to the other, a non-experimental mouse (of the same strain, sex and age) was allowed to explore the equipment for 10 min shortly before tests began. All behavioural testing, unless otherwise stated, was recorded on video and analysed post-hoc and blinded according to ARRIVE criteria (S1 ARRIVE Checklist) [12].

#### Elevated Plus Maze (EPM)

24 hours post injection mice were placed individually in the central square of an elevated plus-maze facing a closed arm, and behaviour was observed for 5 minutes. Time spent in the open arm, number of open arm entries, total arm entries and the latency to enter the first open arm were measured subsequently off-line from video recordings. An animal was considered to have entered an arm when all four legs were out of centre square. EPM testing was carried out during the light phase of the animals light:dark cycle at 350–400 Lux. However, it should be noted this test measures anxiety behaviours and is principally used to measure rodents’ aversion to elevated and open spaces, the ‘closed’ arms have sides but no top and therefore are exposed to the same levels of light as the open arms when the light source is from above [13].

#### Open Field

Animals were subjected to open field testing as described previously [14, 15] using an open-topped, black, rectangular box (50 × 30 × 30 cm) with the floor divided into 10 × 10 cm squares, under light levels of 350–400 Lux. Mice were placed individually in a corner square, and the number of squares entered in a 3 min period was measured post-hoc on video recordings. The number of rears (both front paws off the ground, but not as part of grooming) were also counted. The latency for the mouse to leave (with all four feet) the corner square, and latency to the first rear, were also measured. As the open field is a large inescapable and novel environment, mice tend to show a preference towards the perimeter rather than the central area and thus measuring centre:perimeter ratio provides a relative metric of anxiety [16]. Sick animals also show reduced thigmotaxis during the acute phase of inflammation and it is therefore a good measure of general exploratory behaviour.

#### Forced Swim Test (FST)

Mice were placed individually for 6 min into clear Perspex cylinders (height 25 cm, diameter 20 cm) containing 15 cm of water (23–25°C). The duration of immobility was recorded during the last 4 minutes of the 6 minute testing period. Water was changed after each test. A mouse was considered to be immobile when it floated in an upright position, and made only small movements to keep its head above the water. Climbing activity was considered when it used all its paws to reach the wall of the cylinder and tried to climb. The immobility, climbing and swimming time were scored blindly after the test. The FST shows both face and construct validity when measuring depression-like behaviours [17].

#### Sucrose Preference

On two randomly selected days prior to testing animals were exposed to a single bottle of a 2% sucrose solution for 12 hours overnight to allow them to acclimatise to the taste. During testing, animals were given 12 hours of free choice between two bottles of either 1% sucrose or standard drinking water at 24 hours prior to and 24 hours post-LPS. At the beginning and end of the period the bottles were weighed and consumption calculated. The beginning of the test started with the onset of the dark phase of animals’ cycle. To prevent the possible effects of side-preference in drinking behaviour, the position of the bottles in the cage was switched at 6 hours. No previous food or water deprivation was applied before the test. Percentage preference for sucrose is calculated using the following formula: Sucrose preference = Volume (Sucrose solution)/(Volume (Sucrose solution) + Volume (Water)) × 100. No naïve animals ever exhibited a preference for sucrose of <65% and, accordingly, mice exhibiting a sucrose preference of <65% were defined as showing a depression-like phenotype. This is in accordance with animal models of depression-like behaviour brought on by chronic stress [18, 19].

#### Marble Burying

Mice were placed in clear plastic cages (25cm x 19cm) with a 5cm deep layer of sawdust on which was arranged 15 marbles in a 3 x 5 configuration [20]. Digging behaviour was filmed over 30 minutes noting latency to dig and number of marbles buried at intervals after starting recording. Marble burying shows stereotypical mouse digging behaviour and is often used to demonstrate anxiety, with an increase in marbles buried indicating an anxious state [20]. However, Thomas and colleagues [21] have suggested this is not the case, but rather that it reflects perseverative and repetitive behaviours.

#### (±)-1-(2,5-dimethoxy-4-iodophenyl)-2-aminopropane (DOI)-Induced Head-Twitch Behaviour

Mice received a single dose of DOI (1mg/kg i.p.) and were observed for 20 minutes, during which time the number of head-twitches were counted as a measure of 5-HT_2A_ activity [22].

### 5-HT and 5-HIAA HPLC

Animals were killed by overdose of anaesthetic (sodium pentobarbitone). Blood was removed via cardiac puncture for kynurenine analysis. This was followed by intracardial perfusion of ice-cold 0.9% heparinised-saline, and tissue was rapidly removed from specific CNS regions (cortex, hippocampus, striatum and cerebellum). Samples were analysed using HPLC with electrochemical detection and separated with an ACE column (C18, 3μm, 125 × 3mm + ACE C18 guard, 10 × 3mm run at 35°C). Samples were carried in a mobile phase (12.5% methanol, 130mM NaH_2_PO_4_, 0.85mM Na_2_EDTA, 0.1mM 1-octanesulphonic acid, pH 3.55) pumped at 0.6ml/min (Waters 2695 HPLC Pump). Samples were detected using a glassy carbon electrode held at + 0.75 V (Dionex ED40). The sample content was determined with reference to daily-calibrated standard solutions in 0.06 M perchloric acid (5pmol 5-HT and 5-HIAA). Chromatograms were displayed and analysed using Waters Empower 2 software.

### IDO Activity

Animals were killed as above and tissue was rapidly removed from the same CNS regions as well as from the intestine, specifically a 1 inch portion of the proximal duodenum. Intestinal material was washed prior to homogenization with ice-cold saline. Tissue was weighed and homogenized in cold suspension buffer (250mM sucrose, 50mM Tris-HEPES (pH7.5), 0.2mM EDTA). Homogenate was centrifuged and supernatants analysed for IDO activity. Supernatant was combined 1:1 with incubation medium at 37°C (0.8mM TRP, 40mM ascorbin acid, 20μM methylene blue, 200units/ml catalase in 100mM phosphate buffer) and agitated for 180 minutes. Addition of 30% TCA stopped the reaction and incubation at 50°C for 30 minutes allowed N-formylkynurenine to convert to kynurenine. Samples were centrifuged at high speed and supernatants were analysed by HPLC for the presence of kynurenine.

### Kynurenine HPLC

HPLC was performed as described by Zhang *et al*. [23]. Standard stocks of kynurenine and tryptophan were prepared at 10mM in dilute HCl and diluted as appropriate in dialysis buffer or control plasma for calibration purposes. For CNS dialysates, samples (20μl) were analysed without further treatment; plasma (50μl) was deproteinised with 10 μl 26% perchloric acid, centrifuged at 13,000 g for 10 min at 4°C, and 20 μl used for analysis. Concentrations were determined by HPLC with absorbance detection, using an ACE C18 column, 3μm, 150 x 3 mm and eluent 5% acetonitrile, 15 mM potassium acetate, pH 4.0, flow rate 0.5 ml/min, using a diode array absorbance detector monitored at 360 nm (kynurenine). Time during the initial incubation phase and initial weight of tissue were taken into account and final data was presented as the concentration of kynurenine produced (pmol) per mg of tissue per hour.

### RNA Extraction and Quantitative RT-PCR

RNA extraction was performed as previously described from specifically microdissected snap-frozen brain regions [11, 24]. Specific primers were designed by PrimerDesign as follows: TNF-F GCCTCCCTCTCATCAGTTCTAT; TNF-R TTTGCTACGACGTGGGCTA; IL-1β-F CAACCAACAAGTGATATTCTCCAT; IL-1β-R GGGTGTGCCGTCTTTCATTA; 5-HT_2A_-F CAGGCAAGTCACAGGATAGC; 5-HT_2A_-R TTAAGCAGAAAGAAAATCCCACAG; 5-HTT-F TGCCTTTTATATCGCCTCCTAC; 5-HTT-R CAGTTGCCAGTGTTCCAAGA; IDO-F TGCTTACTCTCTTTTCCCTTCC; IDO-R CATCAGACCTGGTGCTTCA. Quantitative RT-PCR was run using SYBR green based technology (Primer Design Ltd.). Results are expressed as relative-fold expression compared to control animals and corrected to the housekeeping gene, glyceraldehyde 3-phosphate dehydrogenase (GAPDH).

### Statistics

Statistical analysis were performed using GraphPad Prism 5.0 and InVivoStat software using two-way ANOVA and Student’s t-tests, and appropriate post-hoc analysis, as described in the text. Data are presented as mean ±SEM.

## Results

### LPS causes depression-like behaviours at 24 hours

**Fig 1:**
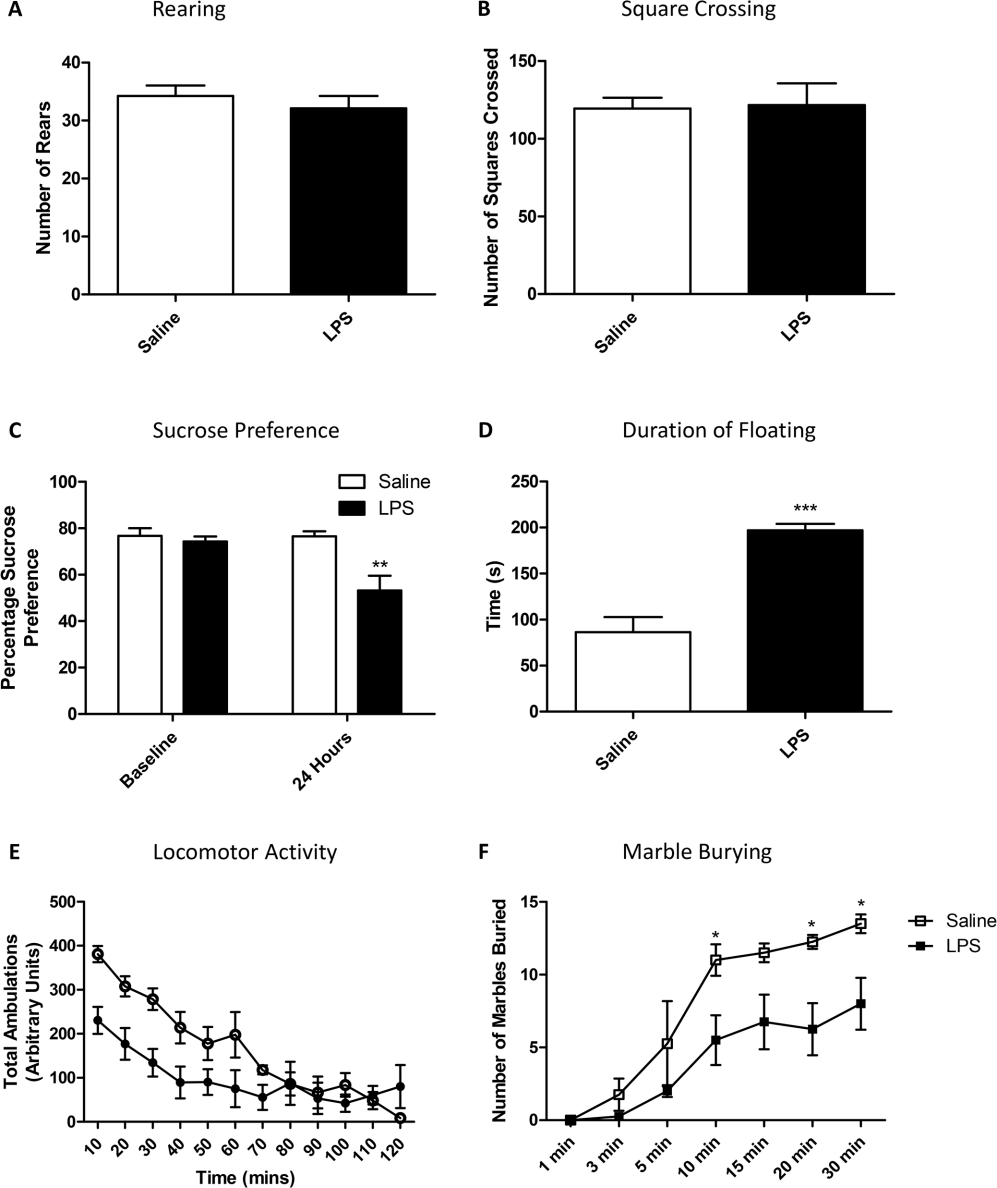
Open field and locomotor activity in saline and LPS treated animals at 24 hours. Animals received a single dose of LPS (0.5 mg/kg) or vehicle 24 hours prior to testing, and were tested using a standard 3-minute open field measuring rearing (A) and square crossing (B). Animals were also tested using a two-bottle sucrose preference test (C) and a forced swim test studying duration of floating behaviour (D), as well as a longer 2 hour locomotor activity study (E) and marble burying test (F). Data are mean ±SEM n = 6; *p<0.05, **p<0.01 and ***p<0.001 compared to saline injected controls.

To determine whether basic exploratory and mood-related behaviours were altered 24 hours after an LPS challenge, animals were subjected to a standard 3-minute open field test, as well as a longer locomotor activity test, to examine both exploratory and anxiety-type behaviours. Open field behaviour was not different in terms of either rearing behaviour (Fig 1A) or distance travelled (Fig 1B) during a 3-minute window. During a longer 2 hour locomotor activity task, all animals slowed down over time, and there was an interaction between LPS-challenge and time but no main effect of LPS (RM-ANOVA time p<0.001 F_11,110_ = 26.90; LPS p = 0.07 F_1,110_ = 3.85; LPS:time p<0.001 F_11,110_ = 5.09; Fig 1E). It should be noted that this dose of LPS is capable of inducing sickness behaviours at an acute time point S1 and S2 Figs) as well as a hepatic acute phase response (S2 Fig). It has been previously shown that peripheral inflammation can cause depression like behaviour in mice [25]. Here, 24 hours after a single LPS challenge, mice showed significant changes in sucrose preference, forced swim behaviours and marble burying. Specifically, after 24 hours LPS-treated animals showed a decrease in sucrose preference to <65% showing main effects of both time after testing and LPS, as well as an interaction (RM-ANOVA time p<0.05 F_1,6_ = 6.51; LPS p<0.01 F_1,6_ = 13.21; LPS:time p<0.05 F_1,6_ = 6.30; Fig 1C). Bonferroni post-hoc tests showed a decrease in sucrose preference in LPS treated animals, compared to saline treated animals (p<0.01). In the forced swim test, floating behaviour reached almost twice saline treated levels in animals receiving LPS (p<0.001; Fig 1D). Finally, marble burying increased across time and this behaviour was significantly decreased in LPS treated animals (RM-ANOVA time p<0.001 F_6,36_ = 33.69; LPS p<0.05 F_1,36_ = 8.71; LPS:time p = 0.053 F_6,36_ = 2.32; Fig 1F) with post-hoc analysis showing significant differences between groups after 10 minutes of testing (Bonferroni post-hoc p<0.05). It should be noted that there was no impairment in the animals’ overall levels of locomotor activity in this test (p = 0.93; S3 Fig).

### Systemic Inflammation does not cause changes in CNS 5-HT levels

**Fig 2:**
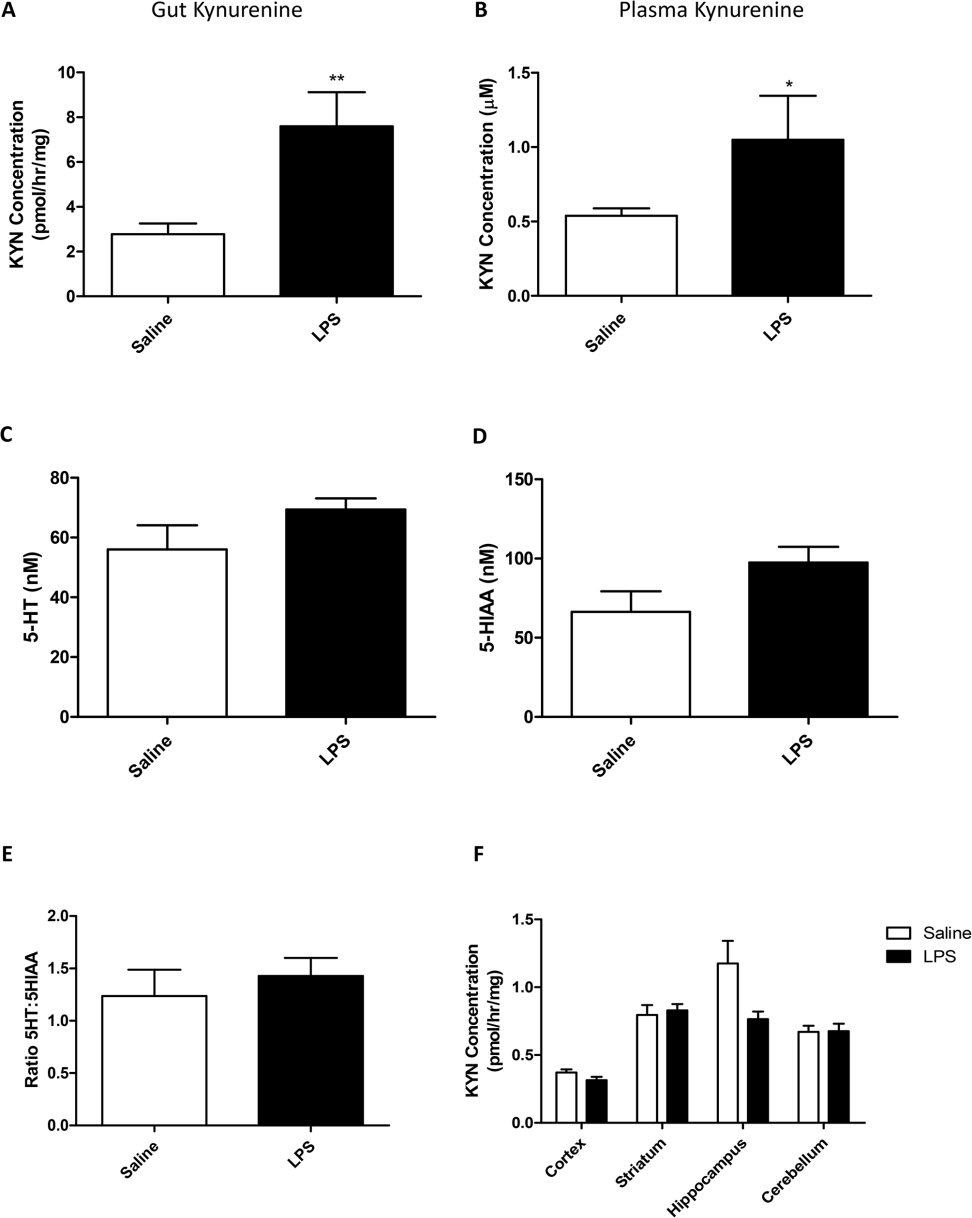
Kynurenine and 5-HT levels in the circulation and tissues of saline and LPS treated animals at 24 hours. Animals received a single dose of LPS (0.5mg/kg) or vehicle 24 hours prior to testing for kynurenine production by IDO in the gut (A) and kynurenine levels in the plasma (B). Whole brains were assessed for 5-HT (C); 5-HIAA (D); and the ratio of 5-HT:5-HIAA (E) as well as kynurenine production by IDO in different brain regions (F). Data are mean ±SEM n = 6; *p<0.05; **p<0.01 and ***p<0.001 compared to saline injected controls.

**Fig 3:**
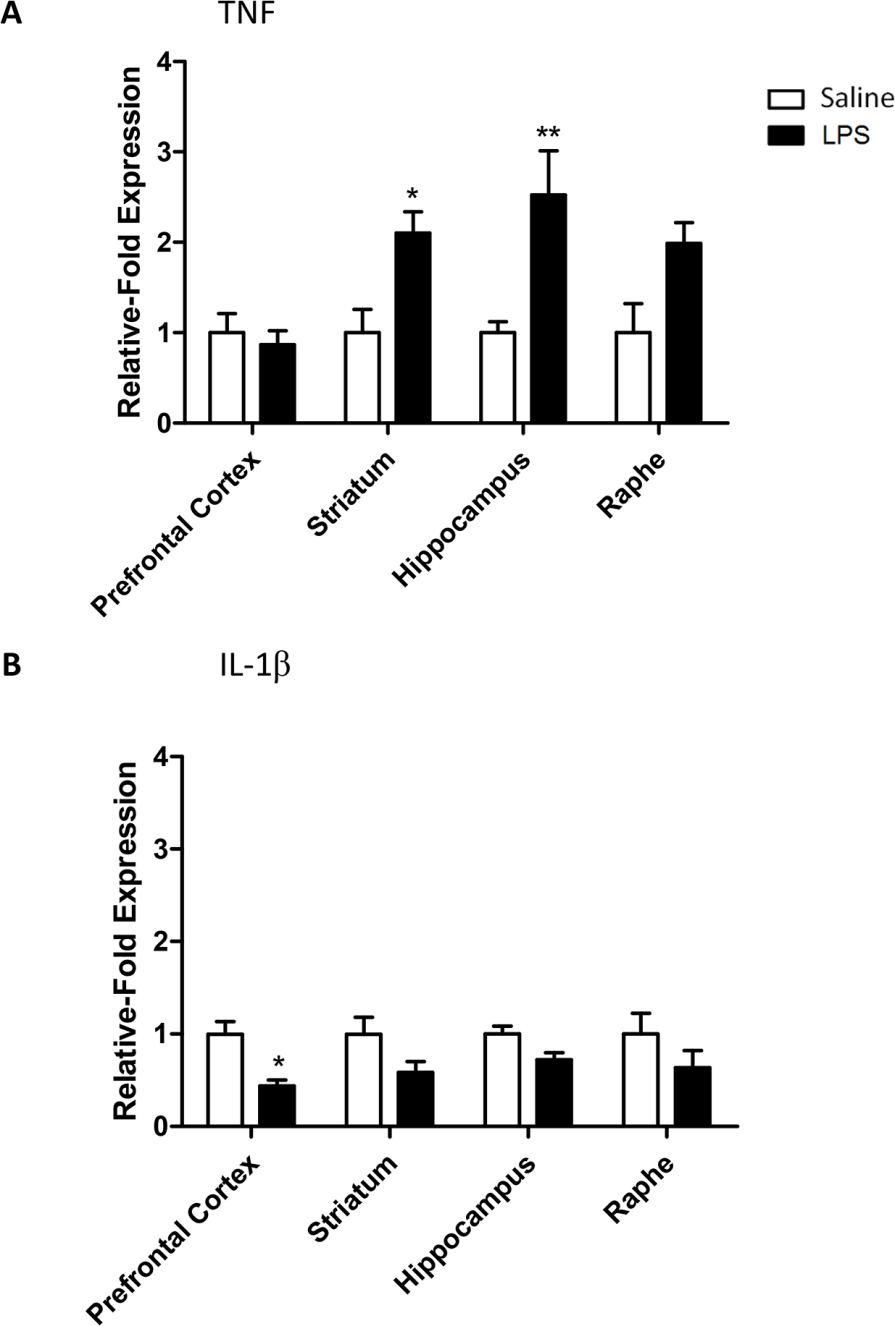
Cytokine mRNA expression in the CNS 24 hours after an LPS challenge. (A) TNF and (B) IL-1β transporter mRNA levels expressed as relative-fold expression compared to saline controls within each region. mRNA expression is normalized to the housekeeping gene GAPDH prior to analysis. Data are mean ±SEM n = 6; *p<0.05 and **p<0.01 compared to saline injected controls.

**Fig 4:**
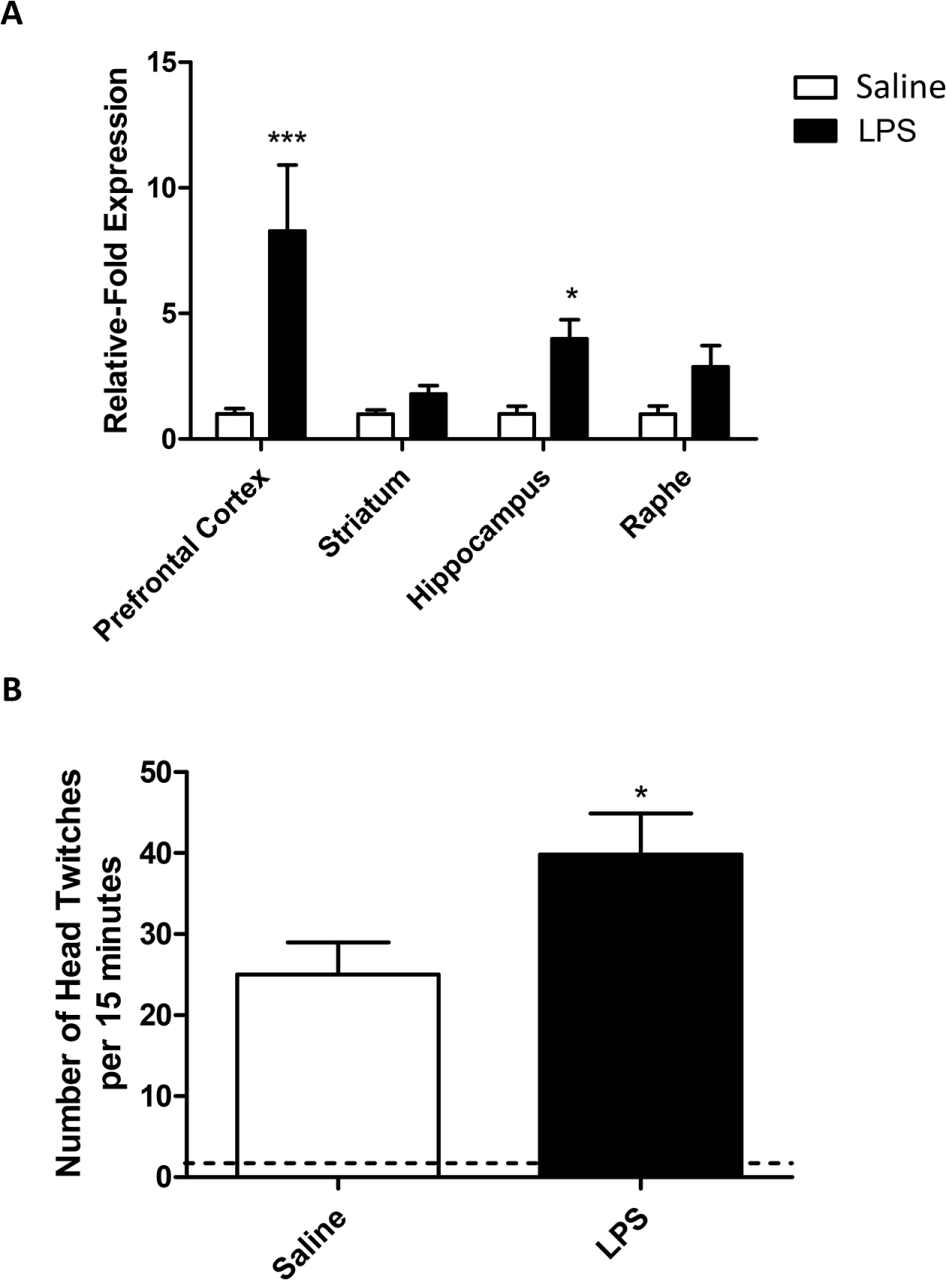
5-HT_2A_ receptor changes after LPS. mRNA expression in the CNS 24 hours after an LPS challenge (A), mRNA levels are expressed as relative-fold expression compared to saline controls within each region. mRNA expression is normalized to the housekeeping gene GAPDH prior to analysis. LPS-induced changes in 5-HT_**2A**_-mediated head-twitch responses (B). Animals received a single dose of LPS (0.5mg/kg i.p.) and were allowed to recover for 24 hours prior to receiving a single dose of DOI (1mg/kg i.p.). Head twitch behaviours were observed over a period of 15 minutes. Dotted line represents baseline (no DOI) data for both LPS and saline treated animals. Data are mean ±SEM n = 6; *p<0.05, **p<0.01 and ***p<0.001 compared to saline injected controls.

Systemic inflammation is known to alter the activity of IDO, which is strongly expressed in the gut and the CNS. This enzyme is known to metabolize tryptophan and thus reduce its availability for 5-HT synthesis. In order to determine whether inflammation affected IDO activity, and as a consequence, 5-HT and kynurenine levels in the CNS, all of the above were measured in plasma, CNS and gut. At 24 hours after an LPS challenge kynurenine concentrations in the gut were significantly increased (Student’s t-test p<0.01; Fig 2A). Concomitantly, plasma levels of kynurenine also increased in LPS-treated animals (p<0.05; Fig 3B). Total brain 5-HT, and its metabolite 5-HIAA were assessed by HPLC 24 hours after a single LPS injection (0.5mg/kg) and a ratio of 5-HT:5-HIAA was used to assess turnover (5-HIAA/5-HT). There was no significant change in either 5-HT or 5-HIAA (Student’s t-test p = 0.16 and 0.10, respectively; Fig 2C and 2D), and also no change in the ratio of 5-HT to 5-HIAA (p = 0.5; Fig 2E). Furthermore, kynurenine levels within specific regions were investigated to determine whether the immune axis within the brain had any effects on tryptophan breakdown. LPS and the region studied both had significant effects on kynurenine accumulation (two-way ANOVA region p<0.001 F_4,50_ = 25.71; LPS p<0.05 F_1,50_ = 4.15; LPS:region p<0.05 F_4,50_ = 3.82; Fig 4D), however, post-hoc analysis revealed no significant effect of LPS in any brain region (Bonferroni p>0.05 in all cases). The lack of change in IDO related activities was backed up by no change in IDO mRNA expression after an LPS challenge, even at acute time points where changes are likely to occur (S4 Fig).

### LPS increases TNF mRNA in the CNS at 24 hours

Bacterial LPS is known to cause a number of systemic effects, including changes in body temperature, which are largely resolved by 24 hours [26]. We wished to determine whether the same was the case for CNS inflammation caused by a systemic challenge. TNF mRNA expression was up-regulated in the striatum, hippocampus and raphe (RM-ANOVA brain region p<0.05 F_3,24_ = 3.34; LPS p<0.001 F_1,24_ = 20.30; LPS:brain region p<0.05 F_3,24_ = 3.34; Fig 3A) with significant increases in the striatum and hippocampus (Bonferroni post-hoc p<0.05 and p<0.01, respectively). Conversely, IL-1β expression was not increased in any brain region but rather fell in the prefrontal cortex (RM-ANOVA brain region p = 0.7 F_3,24_ = 0.36; LPS p<0.001 F_1,24_ = 16.67; LPS:brain region p = 0.7 F_3,24_ = 0.36; Fig 3B).

### Systemic inflammation increases 5-HT_2A_ receptor expression and function

After determining that LPS induces behavioural effects and an increase in central TNF expression, we decided to establish whether the expression of specific 5-HT receptors was altered by peripheral inflammation. 5-HT_2A_ receptor mRNA expression was increased after exposure to a systemic inflammatory challenge and was affected in all brain regions (RM-ANOVA brain region p<0.05 F_3,24_ = 3.77; LPS p<0.001 F_1,24_ = 19.54; LPS:brain region p<0.05 F_3,24_ = 3.77; Fig 4A). 5-HT_2A_ expression in the pre-frontal cortex and hippocampus was significantly increased in LPS treated animals compared to controls (Bonferroni post-hoc p<0.001 and p<0.05, respectively). 5-HT_2A_ receptor mRNA expression was increased after exposure to a systemic inflammatory challenge, we therefore decided to find out whether this had an effect on 5-HT_2A_-mediated behaviours. (±)-1-(2,5-dimethoxy-4-iodophenyl)-2-aminopropane (DOI) induces a head-twitch response in mice after systemic exposure, and this occurs in a 5-HT_2A_-dependent manner [22]. DOI had a significant effect on head-twitch behaviours in animals treated with both saline and LPS, however, this was elevated in animals receiving a single dose of LPS 24 hours before, compared to those receiving DOI alone (p<0.05; Fig 4B).

## Discussion

The acute phase of LPS-induced sickness behaviour is associated with a febrile response, decreased locomotor behaviour and a general reduction in activity. In the next phase of recovery from sickness, usually after 24 hours, animals continue to display depression-like behaviours and fatigue. Here, we sought to characterize the changes in expression of 5-HT related genes associated with these behavioural changes. Together, the data suggest the novel idea that systemic inflammation may alter behaviour by changing the expression of 5-HT receptors, rather than by reducing the availability of 5-HT in the CNS, which is consistent with observations in CFS/ME and depressed patient cohorts [2, 27, 28].

Depression-like behaviour is often evaluated using the sucrose preference test, with depressive anhedonia indicated by a low preference for sweet drinking solutions, as well as decreased activity in the forced swim test [29]. The forced swim test, however, can pose ethical problems when combined with sickness. Introducing rodents to a wet environment while they may be suffering from hypothermia might be considered unnecessary. Here, we have demonstrated that the marble burying test effectively demonstrates reduced activity, which is not associated with reduced locomotor behaviour, without the need to expose the animals to an aversive environment. Considering the issues that patients with mood problems have with initiation and perseverance tasks this may be significant [30].

It is well known that depression and depressive-like behaviours are associated with higher levels of circulating cytokines [31–35]. However, the causal nature of this relationship is currently speculative. Here, we have introduced the circulating cytokines with a systemic immune challenge and therefore any changes in behaviour are likely to be due to the increased levels of inflammation, rather than the other way around. The IDO theory postulates that pro-inflammatory cytokines up-regulate the activity of IDO and therefore deplete the levels of tryptophan available for making 5-HT [36]. Perhaps the most surprising result therefore, was that brain tissue levels of tryptophan breakdown to kynurenine and the levels of 5-HT and 5-HT metabolites were unaltered in the depressive-like late phase of sickness (>24 hours) in these animals. The role of IDO in the breakdown of tryptophan suggests that any circulating factors altering the activity of this enzyme, should alter overall 5-HT metabolism [37–39]. It is clear from the work here that peripheral IDO activity is increased by systemic inflammation and this is reflected in the increased IDO activity in the gut and the increased levels of circulating kynurenine. Others have demonstrated similarly large increases in the periphery [40], however, increases of this magnitude are rarely reflected by similarly large increases in the CNS. These data suggest that while the tryptophan/kynurenine pathway may be involved in inflammation-associated behavioural changes, this is likely to be an entirely peripheral phenomenon. The low n number used here may bias the HPLC results towards a type II error, however, the changes in behaviour and receptor expression were significant after LPS and therefore an increase in n to investigate the peripheral phenomenon theory was beyond the scope of this report. Indeed, a study by Hughes and colleagues [41] has suggested that changes in tryptophan occur independently of changes in IDO and inflammatory cytokines, so it is possible that the alteration in IDO activity has other functions that are unrelated to depression-like behaviours.

Recent research studying behavioural changes in response to inflammatory challenges often focus on the role of specific cytokines in the CNS. Two major pro-inflammatory cytokines are TNF and IL-1. It has been suggested that the only role for TNF in sickness behaviour is to compensate for dwindling IL-1 [9, 42]. In apparent contradiction to this, our data has shown that a systemic challenge with LPS results in significant up-regulation of CNS TNF, with no significant elevations of IL-1. This selective up-regulation of particular cytokine and chemokine subsets has also been observed in human studies using vaccines [43]. The role of TNF, specifically, as a mediator of sickness behaviour, is evident in studies using anti-TNF drugs, which appear to reverse sickness behaviours when they are given systemically [10]. It has also been shown that TNF receptor knockout animals show a distinctly different behavioural phenotype, specifically an anti-depressant phenotype in the forced swim, compared to littermate controls [44], where no such behavioural change has thus far been noted in any of the IL-1 knockout animals (personal communication). Clinically, TNF presents an interesting prospect. Anti-TNF therapy has been shown to be anti-depressant in the absence of a clinical improvement in symptoms [45], whereas, much like the knockout mouse example, no such data exists for anakinra, the IL-1 receptor antagonist mimic. With studies in depressed patients beginning to investigate the potential of inhibiting cytokines in treatment-resistant depression, there is a clear positive role for cytokines such as TNF in the regulation of mood [46]. However, the mechanisms by which TNF mediates changes in behaviour, and its potential role in both sickness and depression, are yet to be explored.

Perhaps the most commanding results here are the changes we have observed in the 5-HT system. In this study, treatment with LPS caused a significant increase in 5-HT_2A_ receptor expression in a number of discrete brain regions. We have also demonstrated that this results in a functional outcome, i.e. there is a significant alteration in 5-HT_2A_-mediated behaviours. This is in accord with our previous studies on acute sickness, where increases in 5-HT_2A_ receptor expression were also associated with functional changes [24]. Our data also indicate that these changes are receptor specific, rather than a general change in 5-HT receptor expressing cells, as we do not see similar changes in other 5-HT receptors, such as 5-HT_1A_ (S5 Fig). The involvement of the 5-HT_2A_ receptor in mood is currently controversial. Studies from the late 80s indicate that receptor expression increases in patients with suicidal depression [47–49], with further data suggesting that this increase may be non-neuronal [50]. In terms of inflammation, and as a consequence sickness behaviour, increases in 5-HT_2A_ receptor expression have been seen in models of inflammatory pain [51], as well as in other studies using LPS [5] indicating that systemic inflammation is capable of regulating this response *in vivo*. The mechanisms by which changes in receptor expression occur were beyond the scope of this study; however, hypotheses can be generated based on existing research. Studies linking the 5-HT_2A_ receptor with inflammation have suggested that its activation may result in the down-regulation of inflammatory cytokines [52], specifically TNF expression [53]. Furthermore, studies in cultured glioma cells have demonstrated an inhibition of inducible nitric oxide synthase (iNOS) by 5-HT_2A_ agonists [54]. Work by Zhang and colleagues [51] demonstrated that the up-regulation of 5-HT_2A_ after inflammation was not co-localized with 5-HT, indicating that it was not in serotonergic neurons. Furthermore, 5-HT_2A_ receptors have been shown to be present on astrocytes and microglia [55]. Therefore, increased 5-HT_2A_ receptor expression after a peripheral inflammatory stimulus could potentially occur in non-neuronal cells for the purposes of potentiating an anti-inflammatory response in the CNS.

Overall, this study has explored the association between late phase LPS-induced depression-like behaviour and the expression of 5-HT related genes. The data presented here demonstrate that inflammation does not appear to regulate CNS tryptophan levels, but rather may mediate depression-like behaviours by altering the expression and function of CNS 5-HT receptors. This could significantly affect the direction of future research into inflammation associated fatigue and mood disorders, and argues for the use of selective 5-HT receptor agonists and antagonists in their treatment.

## Supporting Information

S1 ARRIVE Checklist The ARRIVE (Animal Research: Reporting of In Vivo Experiments) guidelines were developed as part of an NC3Rs initiative to improve the design, analysis and reporting of research using animals–maximising information published and minimising unnecessary studies. This checklist confirms this study adhered to ARRIVE guidelines (S1 ARRIVE Checklist).
(PDF)

S1 Fig Open field and locomotor activity in in saline and LPS treated animals at 24 hours. Animals received a single dose of LPS (0.5 mg/kg) or vehicle 6 hours prior to testing, and were tested using a standard 3-minute open field measuring rearing (A), square crossing (B), as well as in a longer 2 hour locomotor activity test (C). Data are mean ±SEM n = 6.
(PDF)

S2 Fig TNF mRNA expression in the liver 6 hours after an LPS challenge, mRNA levels expressed as relative-fold expression compared to saline controls. mRNA expression is normalized to the housekeeping gene GAPDH prior to analysis. Data are mean ±SEM n = 6; ***p<0.001 compared to saline injected controls.
(PDF)

S3 Fig General locomotor activity in naïve, saline and LPS treated animals at 24 hours during marble burying. Animals received a single dose of LPS (0.5 mg/kg) or vehicle 24 hours prior to testing, and were tested in the marble burying field. Total locomotor activity within the field was assessed as number of arbitrary squares crossed during allotted time periods. A significant increase in squares over time (p<0.001) was found but there was no difference between groups (p = 0.93). Data are mean ±SEM n = 6.
(PDF)

S4 Fig IDO mRNA expression in the CNS at 6 and 24 hours after an LPS challenge, mRNA levels expressed as relative-fold expression compared to saline controls. mRNA expression is normalized to the housekeeping gene GAPDH prior to analysis. Data are mean ±SEM n = 6.
(PDF)

S5 Fig 5-HT_1A_ mRNA expression in the CNS at 6 hours after an LPS challenge, mRNA levels expressed as relative-fold expression compared to saline controls. mRNA expression is normalized to the housekeeping gene GAPDH prior to analysis. Data are mean ±SEM n = 6.
(PDF)
