# Supplementary figures and images for: A Model of Post-Infection Fatigue Is Associated with Increased TNF and 5-HT2A Receptor Expression in Mice
Source: PLoS One. 2015 Jul 6;10(7):e0130643. doi: 10.1371/journal.pone.0130643 (PMC4493081; doi:10.1371/journal.pone.0130643)

**A**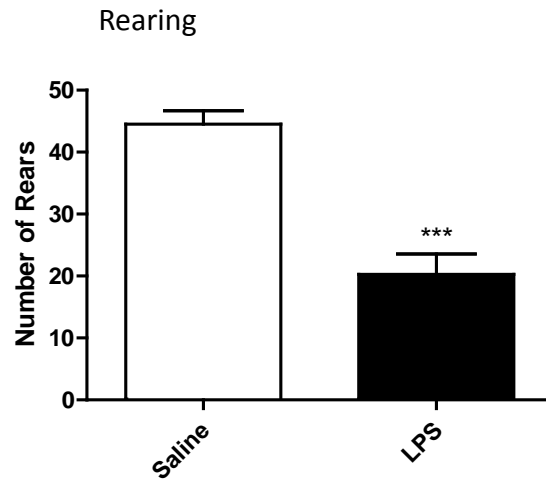**B**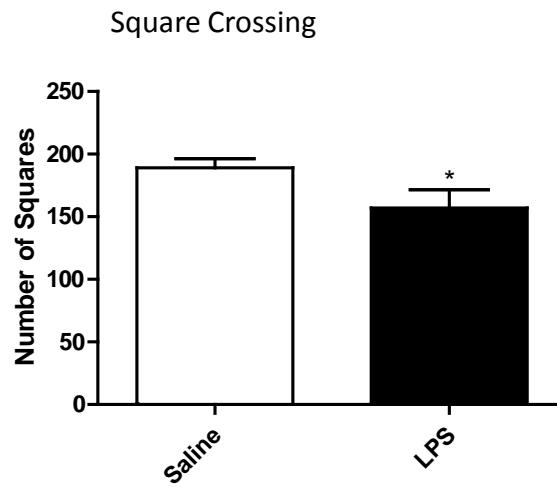**C**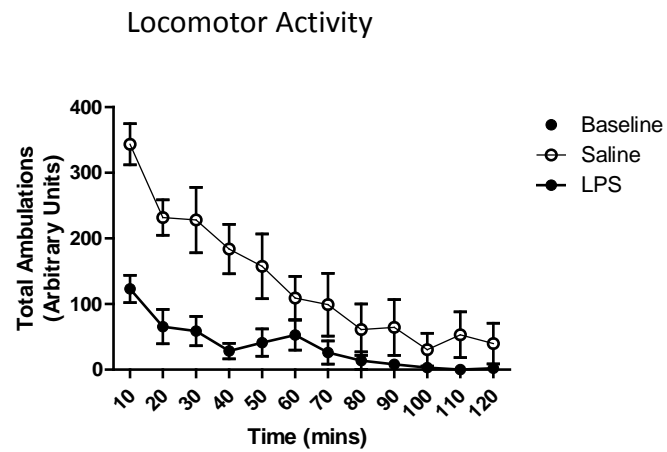

Supplement: S1 Fig — Animals received a single dose of LPS (0.5 mg/kg) or vehicle 6 hours prior to testing, and were tested using a standard 3-minute open field measuring rearing (A), square crossing (B), as well as in a longer 2 hour locomotor activity test (C). Data are mean ±SEM n = 6. (PDF) [file pone.0130643.s002.pdf]

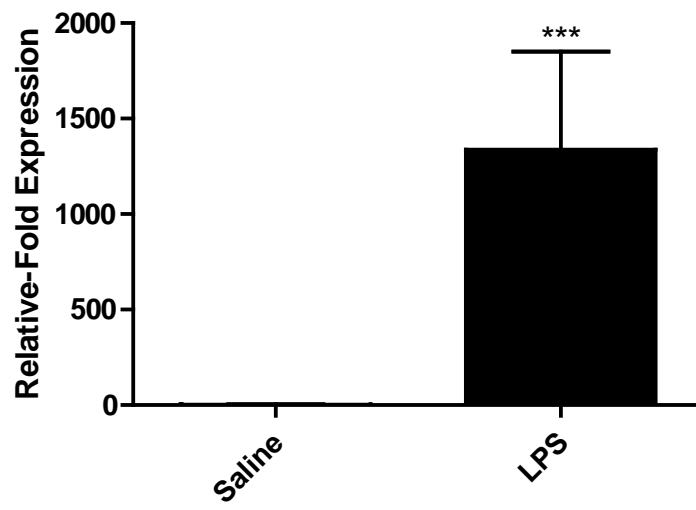

Supplement: S2 Fig — mRNA expression is normalized to the housekeeping gene GAPDH prior to analysis. Data are mean ±SEM n = 6; ***p<0.001 compared to saline injected controls. (PDF) [file pone.0130643.s003.pdf]

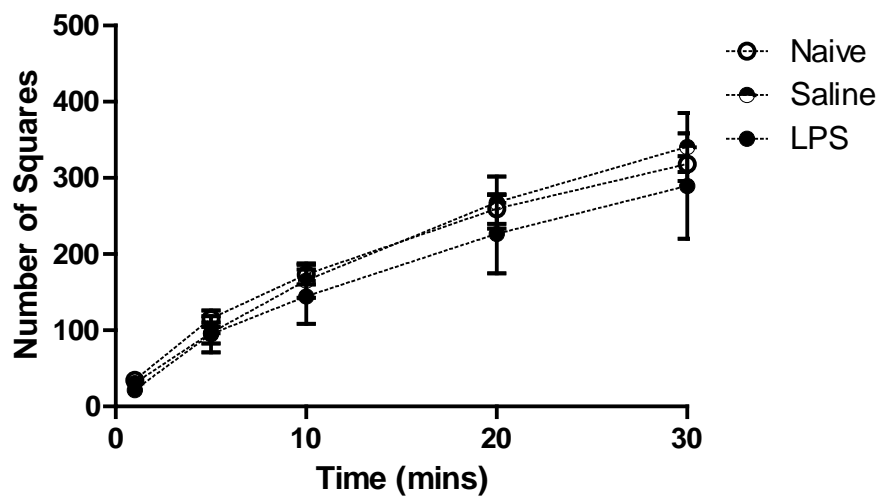

Supplement: S3 Fig — Animals received a single dose of LPS (0.5 mg/kg) or vehicle 24 hours prior to testing, and were tested in the marble burying field. Total locomotor activity within the field was assessed as number of arbitrary squares crossed during allotted time periods. A significant increase in squares over time (p<0.001) was found but there was no difference between groups (p = 0.93). Data are mean ±SEM n = 6. (PDF) [file pone.0130643.s004.pdf]

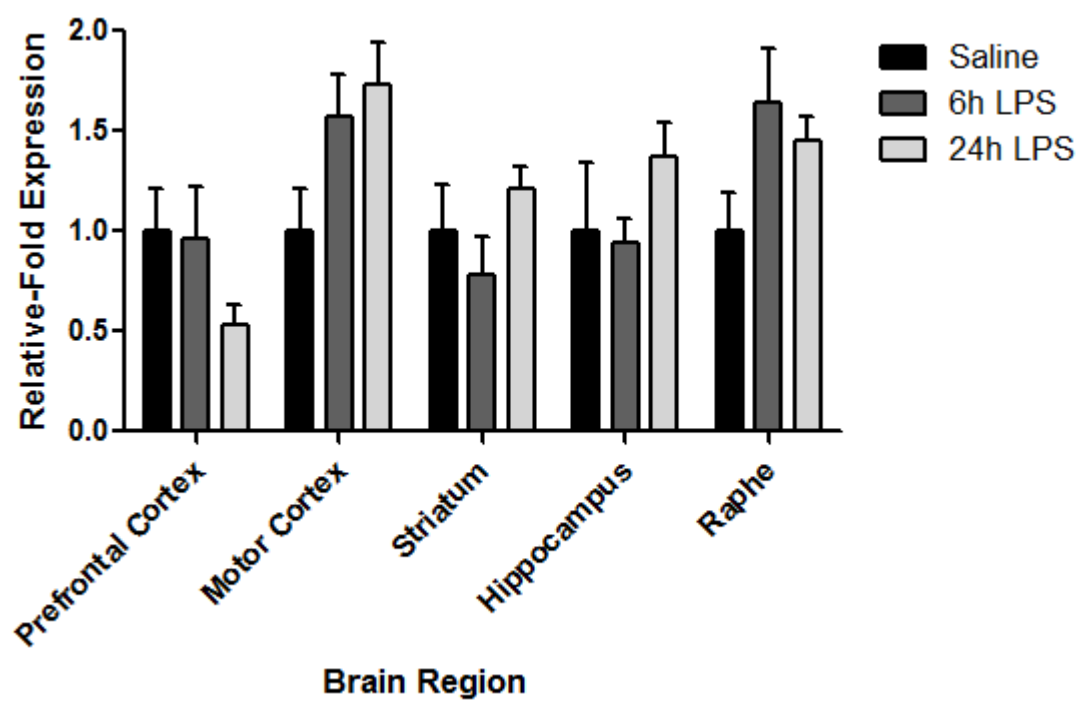

Supplement: S4 Fig — mRNA expression is normalized to the housekeeping gene GAPDH prior to analysis. Data are mean ±SEM n = 6. (PDF) [file pone.0130643.s005.pdf]

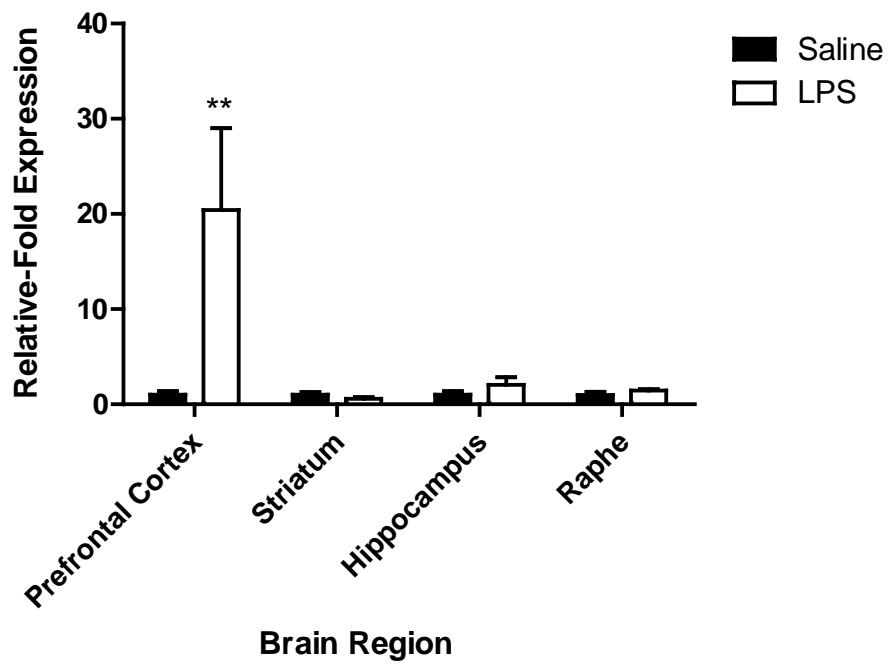

Supplement: S5 Fig — mRNA expression is normalized to the housekeeping gene GAPDH prior to analysis. Data are mean ±SEM n = 6. (PDF) [file pone.0130643.s006.pdf]
